# Genome-Wide Study Updates in the International Genetics and Translational Research in Transplantation Network (iGeneTRAiN)

**DOI:** 10.3389/fgene.2019.01084

**Published:** 2019-11-15

**Authors:** Claire E. Fishman, Maede Mohebnasab, Jessica van Setten, Francesca Zanoni, Chen Wang, Silvia Deaglio, Antonio Amoroso, Lauren Callans, Teun van Gelder, Sangho Lee, Krzysztof Kiryluk, Matthew B. Lanktree, Brendan J. Keating

**Affiliations:** ^1^Division of Transplantation Department of Surgery, University of Pennsylvania, Philadelphia, PA, United States; ^2^Department of Cardiology, University Medical Center Utrecht, University of Utrecht, Utrecht, Netherlands; ^3^Department of Medicine, Division of Nephrology, Vagelos College of Physicians & Surgeons, Columbia University, New York, NY, United States; ^4^Immunogenetics and Biology of Transplantation, Città della Salute e della Scienza, University Hospital of Turin, Turin, Italy; ^5^Medical Genetics, Department of Medical Sciences, University Turin, Turin, Italy; ^6^Department of Hospital Pharmacy, University Medical Center Rotterdam, Rotterdam, Netherlands; ^7^Department of Nephrology, Khung Hee University, Seoul, South Korea; ^8^Division of Nephrology, St. Joseph's Healthcare Hamilton, McMaster University, Hamilton, ON, Canada

**Keywords:** genomics, kidney disease, GWAS, whole exome sequencing analyses, whole genome sequencing

## Abstract

The prevalence of end-stage renal disease (ESRD) and the number of kidney transplants performed continues to rise every year, straining the procurement of deceased and living kidney allografts and health systems. Genome-wide genotyping and sequencing of diseased populations have uncovered genetic contributors in substantial proportions of ESRD patients. A number of these discoveries are beginning to be utilized in risk stratification and clinical management of patients. Specifically, genetics can provide insight into the primary cause of chronic kidney disease (CKD), the risk of progression to ESRD, and post-transplant outcomes, including various forms of allograft rejection. The International Genetics & Translational Research in Transplantation Network (iGeneTRAiN), is a multi-site consortium that encompasses >45 genetic studies with genome-wide genotyping from over 51,000 transplant samples, including genome-wide data from >30 kidney transplant cohorts (n = 28,015). iGeneTRAiN is statistically powered to capture both rare and common genetic contributions to ESRD and post-transplant outcomes. The primary cause of ESRD is often difficult to ascertain, especially where formal biopsy diagnosis is not performed, and is unavailable in ∼2% to >20% of kidney transplant recipients in iGeneTRAiN studies. We overview our current copy number variant (CNV) screening approaches from genome-wide genotyping datasets in iGeneTRAiN, in attempts to discover and validate genetic contributors to CKD and ESRD. Greater aggregation and analyses of well phenotyped patients with genome-wide datasets will undoubtedly yield insights into the underlying pathophysiological mechanisms of CKD, leading the way to improved diagnostic precision in nephrology.

## Introduction

The global prevalence of end-stage renal disease (ESRD) continues to climb. In 2016, 19,301 kidney transplants were performed in the United States, and approximately five times as many were performed worldwide^1^^,^^2^. Due to improvements in surgical techniques, immunosuppression protocols, and clinical management of post-transplant complications, the five-year graft survival rates for kidneys obtained from deceased and living donors reached highs of 75.3% and 85.3%, respectively (Cohen et al.,2006;Serur et al., 2011; Vignolini et al., 2019). However, the prevalence of ESRD cases in the US has continued to rise by ∼20,000 cases per year over the past three decades, creating an increased need for kidney allografts^1^. This increase is believed to be due primarily to worsening diets and other modifiable factors associated with Western lifestyle but also to an increase in the longevity of pre-transplant ESRD cases.

It is well established that genetic factors contribute to the development and progression of specific types of chronic kidney disease (CKD), yet many previous studies have been limited in scope due to small sample sizes and genotyping strategies (Azarpira et al., 2014; Misra et al., 2014; Phelan et al 2014; Parsa et al., 2017; Stapleton et al., 2018). Studies of families with severe phenotypes of diseases, such as Alport’s Syndrome and Fabry Disease, have significantly contributed to the understanding of the genetic characteristics of these conditions (Gillion et al., 2018; Kashiwagi et al., 2018; McCloskey et al., 2018). However, milder forms of these diseases and their role in the development of ESRD have yet to be explored in great depth.

### Genome-Wide Genotyping Arrays

Array based genome-wide genotyping from diverse patient populations facilitates very precise ancestry determination using methods such as principal component analysis (Cai et al., 2013; Li et al., 2015). Genome-wide association studies (GWAS) among patients with CKD have detected both rare and common genetic variants significantly associated with estimated glomerular filtration rate (eGFR) decline and microalbuminuria, some of the strongest predictors of CKD outcomes, despite >80% of GWAS participants having eGFRs in the normal range (Boger et al., 2011a;Boger et al., 2011b;Reznichenko et al., 2012; Gorski et al., 2015; Parsa et al., 2017; Limou et al., 2018).

The findings of genome-wide studies may also provide new therapeutic targets to slow the progression of CKD to ESRD, which may delay or impact the need for transplantation in some patient populations (Wuttke & Kottgen, 2016; Kalatharan et al., 2018). For example, nephropathic cystinosis, a rare autosomal recessive disease, is caused by a 57-kb deletion in the *CTNS* gene in ∼75% of patients of European ancestry and progresses to ESRD if left untreated (Brodin-Sartorius et al., 2012). However, treatment with oral cysteamine by five years of age has been found to significantly decrease the prevalence and delay the onset of ESRD (Brodin-Sartorius et al., 2012). Additionally, at least 38 genes have been associated with the development of genetic focal segmental glomerulosclerosis (FSGS), some of which have been shown to be responsive to glucocorticoid treatment (Rosenberg & Kopp, 2017). GWAS findings can also provide insight into the biology of ESRD, helping to remove diagnostic heterogeneity. The two *APOL1* risk alleles (G1 and G2) found in high frequency in sub-Saharan African populations and strongly associated with FSGS and HIV nephropathy were found to activate protein kinase R, thus inducing glomerular injury and proteinuria (Kopp et al., 2011; Limou et al., 2014; Okamoto et al., 2018). Overall, results from genome-wide screening can enable physicians to provide accurate genetic diagnoses for the primary cause of ESRD, enabling timely and effective therapeutic managemenvwt and aiding in the evaluation of family members as living donors (Snoek et al., 2018).

#### Whole-Exome and Whole-Genome Sequencing

In the last decade, whole-exome sequencing (WES) and whole-genome sequencing (WGS) approaches have been used very successfully to discover and diagnose genetic disorders in a clinical context (Mallawaarachchi et al., 2016; Lata et al., 2018; Warejko et al., 2018;Groopman et al., 2019). WES typically yields sufficient depth of sequencing coverage across ∼95% of nucleotides in coding regions captured and has been used to diagnose rare high penetrant, Mendelian disorders, discover common variants, and identify causal mutations in cancer (Huang et al., 2018; Zhang et al., 2018). WES has recently been implemented as a first-line diagnostic tool in clinical medicine. In a study on fetuses with congenital anomalies of the kidney and urinary tract (CAKUT), pathogenic variants were discovered in 13% of cases (Lei et al., 2017). WES has also been applied to adult-onset CKD and ESRD, in which ∼10% of cases are caused by Mendelian mutations (Wuhl et al., 2014; Lata et al., 2018; Groopman et al., 2019). In a cohort of >3,000 patients with advanced CKD and ESRD ascertained for a clinical trial, WES identified diagnostic variants in 9.3% of patients encompassing 66 monogenic disorders (Groopman et al., 2019). Of the 343 detected variants, 141 (41%) had not been previously reported as pathogenic. Additionally, diagnostic variants were identified in 17.1% of individuals with nephropathy of unknown origin, altering medical management by initiating multidisciplinary care, prompting referral to clinical trials, and guiding donor selection for transplantation (Groopman et al., 2019). However, it should be noted that many CKD studies using WES have struggled to obtain adequate control populations. iGeneTRAiN has a large pool of healthy donors (in kidney and in other organs), which represents a strong advantage for our study designs.

WGS is the most comprehensive approach for the detection of inherited variants due to more complete genome-wide coverage, although there are additional challenges compared to WES. WGS can capture single nucleotide genetic variants, small Insertions and Deletions (Indels), and Copy-Number Variants (Cnvs) throughout the human genome. Although it has a higher cost per sample and can be more difficult to analyze than wes, greater diagnostic yields are evident in patients with negative or inconclusive wes results (Alfares et al., 2018; Lionel et al., 2018). WGS has been shown to identify a diagnostic genetic variant in ∼10–50% of individuals with a suspected genetic disorder, depending on the clinical study population(S-) being screened (van Der Ven et al., 2018; Groopman et al., 2019; Mann et al., 2019).

#### International Genetics and Translational Research in Transplantation Network

Despite technological advances that enable research to be carried out on a genome-wide scale, many studies have been hindered by small sample sizes in single transplant sites, as well as the vast number of complex donor and recipient clinical covariates and disease-related phenotypes observed in transplantation. The International Genetics & Translational Research in Transplantation Network (iGeneTRAiN) is a multi-site consortium that encompasses >45 genetic studies with ∼51,210 solid-organ transplant subjects (International Genetics and Translational Research in Transplantation Network (iGeneTRAiN), 2015). The iGeneTRAiN consortium aims to discover and validate solid organ transplant related genetic factors and post-transplant complications, including primary disease, disease recurrence, drug- and cardio-metabolic related adverse events, and different forms of allograft rejection (International Genetics and Translational Research in Transplantation Network (iGeneTRAiN), 2015). Of the iGeneTRAiN samples, 54% (n = 28,015) are from kidney transplant cohorts and include 17,742 (63.3%) recipients and 10,273 (36.7%) donors. The genotyped donor DNA provides control samples for all iGeneTRAiN studies, a large advantage over previously published genetic studies.

The iGeneTRAiN consortium designed and developed a genome-wide genotyping array, the “TxArray,” which was enriched with content relating to known or putative transplant-specific genetic associations (Li et al., 2015). The TxArray version 1 contains ∼782,000 genetic markers, with tailored transplant-specific content to capture variants across *HLA*, *KIR*, loss-of-function, pharmacogenomic, and cardio-metabolic loci. The array also contains extensive overlap with the UK Biobank Axiom^®^ Array and the Axiom Biobank Genotyping Array, enabling future joint studies or meta-analyses using conventional, hypothesis-free GWAS approaches (Li et al., 2015).

**Figure 1:**
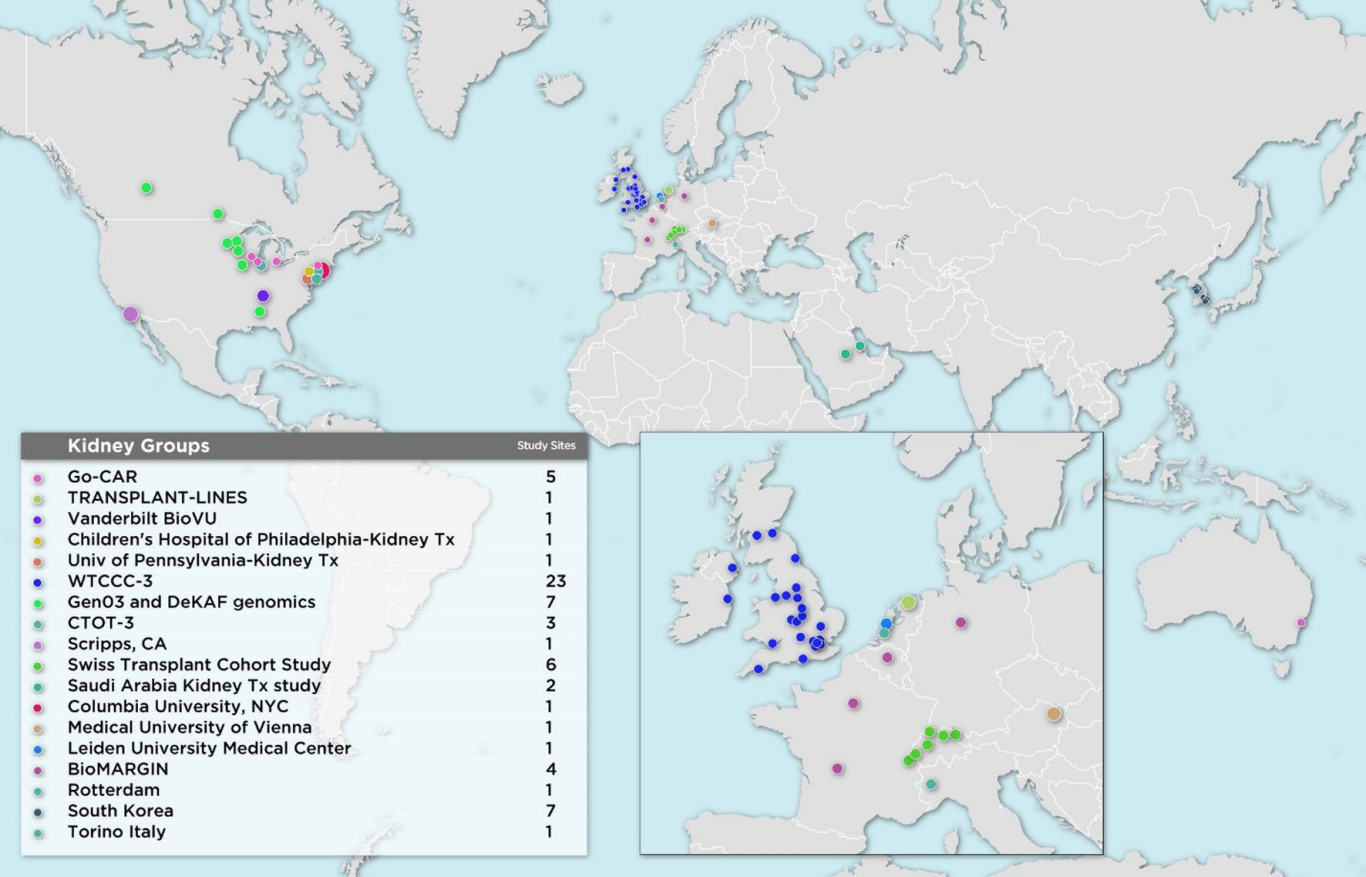
Geographical Map of Current iGeneTRAiN Kidney Sites and Sub-sites.

The first wave of iGeneTRAiN kidney cohorts had a wide geographic representation with participants from various sites in the United States, Canada, Australia, The Netherlands, United Kingdom, and Ireland, including both adult and pediatric sites (**Figure 1**). Over the past few years, many genetic discoveries have been made within the iGeneTRAiN cohorts related to kidney, heart, liver, and lung transplants (Oetting et al., 2016; Greenland et al., 2017; Shaked et al., 2017; Hernandez-Fuentes et al., 2018; Oetting et al., 2018; Snoek et al., 2018; Oetting et al., 2019; Reindl-Schwaighofer et al., 2019). The Wellcome Trust Case Control Consortium (WTCCC) carried out the first large-scale GWAS with both kidney transplant donor and recipient DNA with the goal of identifying genetic variants, in addition to the *HLA* regions, that significantly contribute to long- and/or short-term renal allograft survival (Hernandez-Fuentes et al., 2018). No non-*HLA* signals were observed at genome-wide significance in this initial study, illustrating the need for harmonization of larger, well-phenotyped kidney transplant cohorts. In addition to the previously discovered common loss-of-function variant CYP3A5*3 allele (rs776746), the Deterioration of Kidney Allograft Function (DeKAF) Trial identified two *CYP3A5* variants, rs10264272 and rs41303343, and one *CYP3A4* variant, rs35599367, that explain additional portions of variance observed in dose-adjusted tacrolimus (TAC) through blood concentrations for African American (AA) and European ancestry (EA) kidney transplant recipients, respectively (Dai et al., 2006; Jacobson et al., 2011; Oetting et al., 2016; Oetting et al., 2018). These findings illustrate the utility of genome-wide studies when determining immunosuppression therapy regimens post-transplant, potentially contributing to improvements in renal allograft survival. Another iGeneTRAiN study showed that GWAS performed in nontransplant settings can predict post-transplant complications. Polygenic risk scores calculated from non-melanoma skin cancer (NMSC) GWAS in the general population predicted risk of and time to post-transplant NMSC and added additional predictive value beyond that explained by clinical variables (Stapleton et al., 2019).

#### Ongoing iGeneTRAiN Kidney Genome-Wide Studies

Recently, additional kidney transplant cohorts from Austria, Belgium, Germany, France, Italy, The Netherlands, Saudi Arabia, South Korea, Switzerland, and additional United States sites have joined iGeneTRAiN. This greatly increases ancestral diversity of recipients and donors, as well as statistical power to detect transplant related genetic variants that impact primary disease and transplant outcomes (**Figure 1**). Our large sample sizes are enabling us to investigate both donor and recipient characteristics that effect ESRD cause, treatment, and transplant-related outcomes.

Where available, we obtained formal clinical diagnoses of primary cause of ESRD, organized into disease categories of diabetic, arteriopathic, glomerular, acute kidney injury, infective and obstructive nephropathy, congenital, familial, toxic nephropathy, and malignancies, for all iGeneTRAiN kidney cohort subjects (**Table 1**). With these datasets, we are working to increase our understanding of the genetic underpinnings of ESRD and primary disease through single nucleotide polymorphism (SNP) based GWAS, copy number variant (CNV) screening, donor-recipient properties, allogenicity, and transplant outcomes.

**Table 1:** Primary cause of ESRD in iGeneTRAiN kidney cohorts.

Primary Cause of ESRD	University of Pennsylvania	Transplant-LINES	BioMARGIN^‡^	Swiss Transplant Cohort Study^†^	WTCCC-3^†^	Go-CAR	Columbia Transplant Biobank	Torino Transplant Cohort^‡^	Rotterdam^‡^	DeKAF*	Gen03*	Scripps	Leiden*	Vienna	Vanderbilt	Seoul^†,‡^
**Diabetic nephropathy**	310 (26.8%)	51 (4.0%)	115 (8.5%)	116 (7.4%)	186 (7.3%)	219 (37.2%)	176 (15.6%)	22 (3.3%)	– (17.0%)	553 (28.5%)	178 (22.8%)	–	22 (25.0%)	113 (12.8%)	–	240 (20.9%)
**Arteriopathic renal disease**	202 (17.5%)	104 (8.2%)	67 (4.9%)	183 (11.6%)	111 (4.4%)	109 (18.5%)	123 (10.9%)	36 (5.4%)	– (13.0%)	132 (6.8%)	43 (5.5%)	–	–	88 (10.0%)	–	244 (21.3%)
**Glomerular disease**	340 (29.4%)	401 (31.6%)	329 (24.2%)	433 (27.5%)	482 (19.0%)	111 (18.9%)	431 (38.3%)	198 (29.7%)	– (20.0%)	446 (23.0%)	226 (29.0%)	–	14 (15.9%)	296 (33.6%)	–	251 (21.9%)
**AKI**	3 (0.3%)	1 (0.1%)	3 (0.2%)	0 (0.0%)	8 (0.3%)	0 (0.0%)	5 (0.4%)	2 (0.3%)	– (4.0%)	–	–	–	–	4 (0.5%)	–	0 (0.0%)
**Infective and obstructive nephropathy**	31 (2.7%)	107 (8.4%)	79 (5.8%)	85 (5.4%)	203 (8.0%)	22 (3.7%)	17 (1.5%)	87 (13.1%)	– (6.0%)	–	–	–	–	56 (6.4%)	–	6 (0.5%)
**Congenital diseases**	19 (1.6%)	68 (5.4%)	57 (4.2%)	40 (2.5%)	26 (1.0%)	14 (2.4%)	58 (5.2%)	31 (4.7%)	– (3.0%)	–	–	–	–	18 (2.1%)	–	0 (0.0%)
**Familial hereditary renal diseases**	143 (12.4%)	235 (18.5%)	245 (18.0%)	327 (20.8%)	361 (14.2%)	67 (11.4%)	145 (12.9%)	110 (16.5%)	– (14.0%)	308 (15.9%)	123 (15.8%)	–	52 (59.1%)	147 (16.7%)	–	25 (2.2%)
**Toxic nephropathy**	2 (1.7%)	15 (1.2%)	19 (1.4%)	46 (2.9%)	16 (0.6%)	18 (3.1%)	31 (2.8%)	9 (1.4%)	– (1.0%)	–	–	–	–	25 (2.8%)	–	4 (0.4%)
**Neoplasms**	5 (0.4%)	6 (0.5%)	21 (1.5%)	0 (0.0%)	20 (0.8%)	2 (0.3%)	6 (0.5%)	5 (0.8%)	– (3.0%)	–	–	–	–	14 (1.6%)	–	0 (0.0%)
**Other disease**	50 (4.3%)	59 (4.6%)	64 (4.7%)	344 (21.9%)	165 (6.5%)	12 (2.0%)	9 (0.8%)	6 (0.9%)	– (10.0%)	432 (22.3%)	166 (21.3%)	–	–	17 (1.9%)	–	0 (0.0%)
**ESRD of unknown etiology**	29 (2.5%)	168 (13.2%)	161 (11.8%)	0 (0.0%)	0 (0.0%)	14 (2.4%)	125 (11.1%)	160 (24.0%)	– (9.0%)	–	–	–	–	100 (11.4%)	–	185 (16.1%)
**Missing information**	4 (0.4%)	56 (4.4%)	200 (14.7%)	0 (0.0%)	966 (38.0%)	0 (0.0%)	0 (0.0%)	0 (0.0%)	– (0.0%)	67 (3.5%)	44 (5.6%)	–	–	2 (0.2%)	–	193 (16.8%)
**Total**	**1156**	**1271**	**1360**	**1574**	**2544**	**588**	**1126**	**666**	**–**	**1938**	**780**	**–**	**88**	**880**	**–**	**1148**

*Data includes Caucasian only ancestry. ^‡^Recently added iGeneTRAiN kidney cohort.

^†^National level iGeneTRAiN kidney cohort. See **Supplementary Table 1** for full breakdown of primary cause of ESRD.

### Copy Number Variant Screening in iGeneTRAiN Cohorts

Genome-wide genotyping arrays are well established as an effective means for identification of known and novel CNVs (Sallustio et al., 2015; Ai et al., 2016; Verbitsky et al., 2019). CNV screening within iGeneTRAiN subjects is of major interest for both assessing the genetic architecture of primary disease and for allogenicity studies. iGeneTRAiN has developed an extensive loss-of-function (LoF) pipeline which includes haplotype phasing of over 10 million directly genotyped and imputed variants. We are particularly interested in two copy LoF (by single-nucleotide variants and/or CNVs) and integration of one or two copy LoF variants for donor-recipient interaction analyses, for association with time-to-rejection and graft loss events (International Genetics and Translational Research in Transplantation Network (iGeneTRAiN), 2015).

CNV screening in *a priori* regions for primary disease has been performed in iGeneTRAiN cohorts. For example, we performed CNV screening in patients with nephronophthisis (NPH), the most common genetic cause of ESRD in children and often caused by homozygous *NPHP1* full gene deletions (Levy and Feingold, 2000; Hildebrandt, 2010; Wolf & Hildebrandt, 2011). In iGeneTRAiN, we previously examined this region in a subset of iGeneTRAiN studies for adult-onset ESRD (n = 5,606 patients). Of the subjects analyzed, 26 patients showed homozygous *NPHP1* CNV deletions. Interestingly only 12% of these patients were previously diagnosed as having NPH and many presented with ESRD later in adulthood (Snoek et al., 2018). Thus, using the two copy gene loss of *NPHP1* from GWG arrays to ascertain NPH status and examine NPH-related information in iGeneTRAiN studies, including accuracy of case-ascertainment and age-of-onset, shows a strong proof-of-principle for use in other high penetrant autosomal recessive/dominant cases, and the need for further sequencing for rare single-nucleotide variants in adult-onset ESRD patients. Furthermore, in a recent genome-wide analysis of CNVs in almost 3,000 cases of CAKUT, 45 distinct, known genomic disorders at 37 independent genomic loci were identified in 4% of CAKUT cases, and novel genomic disorders were found in an additional ∼2% of cases (Verbitsky et al., 2019). Genome-wide genotyping and imputation using large whole-genome sequencing (WGS) datasets, such as the 1000 genomes project (1KGP), typically cannot identify variants in the most common ancestral populations to a minor allele frequency (MAF) of <0.005, yet it is often possible to identify rare CNVs using monomorphic or SNP based probes across loci.

**Figure 2:**
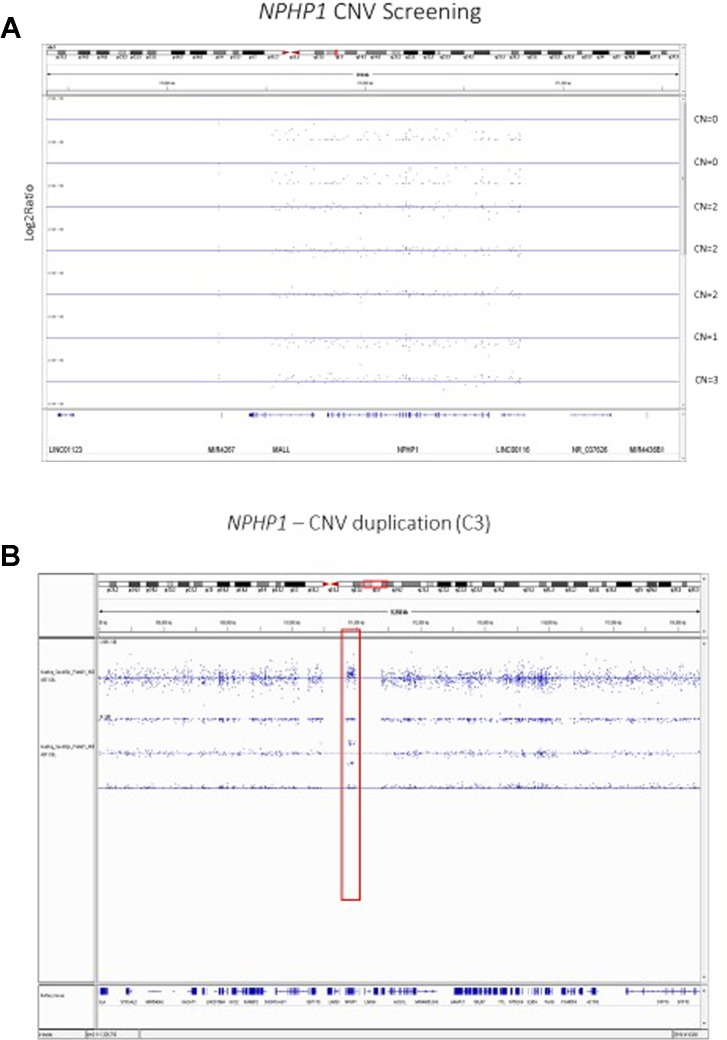
**(A)** Copy Number Variant Region Analyses of the *NPHP1* locus using the Axiom Analysis Suite 4.0 to call diploid state (CN=2), one copy deletion state (CN=1), two copy deletion state (CN=0), and duplication state (CN=3). The x-axis shows the chromosomal location on Chromosome 2 while the y-axis shows the standard Log2Ratio intensity. **(B)** In depth illustration of the duplication (CN=3) state.

Our previous analyses of the Axiom TxArray genome-wide genotyping data was primarily limited to approximately 2,000 *a priori* CNV regions of interest that had specific probes designed onto the TxArray. Initial analyses used an adaption of the BRLMM-P CNV algorithm adapted from algorithms previously used to cluster genotypes across many samples (Yeung et al., 2008). However, BRLMM-P could only identify up to three clusters and thus was only able to detect 0, 1, and 2 copy deletions. The newer Axiom Analysis Suite 4.0 software allows streamlined, targeted, and *de-novo* whole-genome CNV region analysis^3^. A major advantage of the newer software is the ability to detect duplications as well as 0, 1, and 2 copy deletions (**Figure 2**).

## Discussion

Genome-wide genotyping studies have become very affordable and streamlined. However, large sample sizes, on the order of 10,000–100,000, are needed in order to detect both rare variants with large contributions and common variants with minor contributions to a specific phenotype(s) (Korte and Farlow, 2013). While it is very important to bolster statistical power to detect genetic underpinnings of transplant-related phenotypes by aggregating similar cohorts, great caution must be exercised when combining genotyping and phenotyping datasets, especially as transplant study covariates are very complex and can vary greatly by era and geographical region. iGeneTRAiN does have a unified quality control/quality assurance GWAS pipeline, including adjustment for population-based stratification (International Genetics and Translational Research in Transplantation Network (iGeneTRAiN), 2015). Association study analyses do adjust for all known/available study covariates, including patient demographics and clinical characteristics, and we adjust for each transplant site alone to look for confounders. Genome-wide genotyping arrays are generally poor at detecting rarer frequency pathogenic variants, with the exception of medium to large CNVs. Significant advances in genomic technologies and the decreasing cost of WES/WGS efforts over the past several years have made it increasingly feasible to carry out better designed genome-wide studies in a clinical environment (Gumpinger et al., 2018). However, there are still significant advantages to having genome-wide genotyping array datasets, as rigorous quality control and quality assurance measures are generally performed on the original DNA, and gender, ancestry, and *HLA* (amino acid imputation) concordance checks can be performed before progressing to WES or WGS pipelines for deeper genetic characterization. GWAS are able to provide insight into genetic risk scores and pathogenic CNVs, as genome-wide variants are covered in conventional genome-wide genotyping arrays (Sampson and Juppner, 2013; Li et al., 2015 Marigorta et al., 2018; Snoek et al., 2018; Stapleton et al., 2019). For example, a meta-analysis across 36 articles identified three genetic variants that are significantly associated with new onset diabetes after transplantation (NODAT), all of which are also known risk factor variants for Type 2 diabetes. The integration and analysis of large and complex multi-omic datasets has been demonstrated in a number of recent high impact publications, which in general increase, by approximately 10-fold, the statistical power to detect and illustrate functional variants (Chen et al., 2012; Piening et al., 2018; Schüssler-Fiorenza Rose et al., 2019; Zhou et al., 2019). iGeneTRAiN genomic data can be integrated with results from proteome-, metabolome-, and transcriptome-wide transplant studies to further characterize clinical risks and allow for personalized treatments, as a number of iGeneTRAiN studies have multi-omic datasets/samples (International Genetics and Translational Research in Transplantation Network (iGeneTRAiN), 2015).

The advent of single-cell RNA sequencing (scRNASeq) has yielded major insights into the biology of CKD. Expression quantitative trait loci (eQTL) atlases have been generated for glomerular and tubular compartments from human kidney cells. Integrating results from genome-wide studies of CKD with eQTL from scRNAseq as well as known regulatory region maps has been shown to identify novel CKD genes (Qiu et al., 2018). The Human Cell Atlas project is a major international initiative which aims to create comprehensive reference maps of all human cells to gain fundamental insight into the understanding of human health and will undoubtedly aid in the diagnoses and surveillance of a range of diseases (Regev et al., 2017).

### Future of iGeneTRAiN Kidney Cohorts Analyses

As the population of kidney transplant recipients and donors continues to grow in the iGeneTRAiN consortium and as post-transplant outcomes accrue, we will be able to further increase our knowledge of the genetic underpinnings of ESRD, primary disease, and post-transplant outcomes, such as acute rejection and graft loss. These sequencing approaches may provide additional insight into donor-recipient (D-R) interactions that influence graft outcomes. Although it is well established that allelic matches across *HLA* loci impact clinical outcomes post-transplant, there is a paucity of genome-wide research conducted to identify donor-recipient interactions independent of *HLA* (Thorsby, 2009; Chan-On and Sarwal, 2016;Stapleton et al., 2018). One recent iGeneTRAiN kidney D-R study showed decreased allograft survival of recipients with increased D-R kidney transmembrane non-synonymous SNPs (nsSNPs). We further demonstrated that we could detect alloantibodies against customized amino-acid peptides designed with a number of these kidney transmembrane nsSNPs using sera from these patients (Reindl-Schwaighofer, et al., 2019). Finally, data from all solid-organ transplant studies in the iGeneTRAiN consortium will be utilized in cross-organ studies in order to gain additional insight into the genetics of acute rejection, allograft/patient survival, and pharmacogenomic outcomes.

## Ethics Statement

All data used in this publication was collected in accordance with local IRB stipulations.

## Author Contributions

CF, MM, JS, FZ, LC, CW, SD, AA, TG, SL KK, ML and BK all provided data relating to their respective cohorts and all read and provided feedback on the manuscript. BK, MM, and CF performed CNV analyses.

## Funding

Support was received from the Philadelphia Gift-of-Life Organ Procurement Organization.

## Conflict of Interest

The authors declare that the research was conducted in the absence of any commercial or financial relationships that could be construed as a potential conflict of interest.

## Footnotes

1 https://www.usrds.org

2 http://www.transplant-observatory.org

3 https://www.thermofisher.com
